# Genetic vulnerability of exposures to antenatal maternal treatments in 1– to 2-month-old infants

**DOI:** 10.1111/infa.12398

**Published:** 2021-05

**Authors:** Kristina Denisova

**Affiliations:** 1 Sackler Institute for Developmental Psychobiology, Columbia University College of Physicians and Surgeons, New York, NY, USA; 2 Department of Psychiatry, Columbia University College of Physicians and Surgeons, New York, NY, USA; 3 Division of Developmental Neuroscience, New York State Psychiatric Institute, New York, NY, USA; 4 Biobehavioral Sciences Department, Teachers College Columbia University, New York, NY, USA

## Abstract

The growth and maturation of the nervous system are vulnerable during pregnancy. The impact of antenatal exposures to maternal treatments, in the context of genetic vulnerability of the fetus, on sensorimotor functioning in early infancy remains unexplored. Statistical features of head movements obtained from resting-state sleep fMRI scans are examined in 1- to 2-month-old infants, both those at high risk (HR) for autism spectrum disorder (ASD) due to a biological sibling with ASD and at low risk (LR) (*N* = 56). In utero exposures include maternal prescription medications (psychotropic Rx: *N* = 3_HR_; *N* = 5_LR_ vs. non-psychotropic Rx: *N* = 11_HR_; *N* = 9_LR_ vs. none: *N* = 11_HR_; *N* = 16_LR_), psychiatric diagnoses (two or more Dx_2_: *N* = 5_HR_; *N* = 1_LR_; one Dx_1_: *N* = 4_HR_; *N* = 5_LR_; no Dx: *N* = 12_HR_; *N* = 19_LR_), infections requiring antibiotics (infection: *N* = 5_HR_; *N* = 8_LR_; no infection: *N* = 20_HR_; *N* = 22_LR_), or high fever (fever: *N* = 2_HR_; *N* = 2_LR_; no fever: *N* = 23_HR_; *N* = 27_LR_). Movements with significantly higher variability are detected in infants exposed to psychotropics (e.g., opioid analgesics) and those whose mothers had fever, and this effect is significantly worse for infants at HR for ASD. Movements are significantly less variable in HR infants with non-psychotropic exposures (e.g., antibiotics). Heightened number of psychiatric or mental health conditions is associated with noisier movements in both risk groups. Genetic vulnerability due to in utero exposure to maternal treatments is an important future approach to be advanced in the field of early mind and brain development.

## 1 | INTRODUCTION

The fetus is vulnerable to maternal treatments during pregnancy. Pharmacological substances (medications such as opioid analgesics and antidepressants) in the maternal bloodstream can cross the placenta, enter the fetal blood circulation (Hudak & Tan, 2012), and bind to the receptors in the developing fetus. After birth, withdrawal symptoms, termed the neonatal abstinence syndrome (NAS), may occur (Hudak & Tan, 2012). NAS is characterized by disturbances in the functioning of the autonomic, peripheral, and central nervous system, including increased wakefulness and tremors (Hudak & Tan, 2012). Late pregnancy (third trimester) exposures to psychotropics, in particular opioid analgesics, are associated with withdrawal symptoms of NAS (Desai et al., 2015). (Note that opioid (μ) receptors are concentrated in the central nervous system (CNS) and gastrointestinal tract (Hudak & Tan, 2012), which may account for NAS symptoms in infants exposed later in the pregnancy.) Early pregnancy exposure to opioids is associated with birth defects including heart defects (Broussard et al., 2011; Lind et al., 2017), potentially due to dysregulation of growth during organogenesis (Zagon et al., 1999, 2002) (cf. (Broussard et al., 2011)). Moreover, in utero exposures to antidepressants, such as serotonin selective reuptake inhibitors (SSRI), are associated with poorer NAS scores after birth, and elevated NAS scores are associated with poorer fine motor development at 6 months of age (Galbally et al., 2017) and worse motor outcomes in infants (de Vries et al., 2013).

Maternal drugs can also evoke functional changes at the placenta (Sachdeva et al., 2009) by constricting placental blood vessels and reducing blood supply. This process would diminish delivery of nutrients to the developing fetus and appears to be particularly harmful during early pregnancy. Indeed, moderate maternal nutrient restriction has wide-ranging effects on the features of the primate fetal brain, including adverse neuronal and glial maturation (Antonow-Schlorke et al., 2011). In the context of treatment for illicit drugs of abuse, (Walhovd et al., 2012) found altered myelination in infants of methadone-maintained mothers. However, it is important to consider this evidence in the context of the possible role of the severity of underlying psychiatric diagnoses of the mother.

Vulnerability may also be due to indirect consequences of treatments such as antibiotics (which can also cross the placenta; (Pacifici, 2006)). Here, it is the bacterial cell wall which may cross the placenta and trigger undesired biological processes in the fetus, including atypically increased cell proliferation and behavioral deficits as shown in a murine animal model (Humann et al., 2016). In humans, in utero antibiotic exposures are associated with longer-term consequences including sleep and attention-deficit/hyperactivity disorders (ADHD) in childhood (Lavebratt et al., 2019). Concerns about the consequences of antibiotic use during pregnancy for the newborn have also been raised in the context of fetoplacental microbiome (Kuperman & Koren, 2016).

**FIGURE 1:**
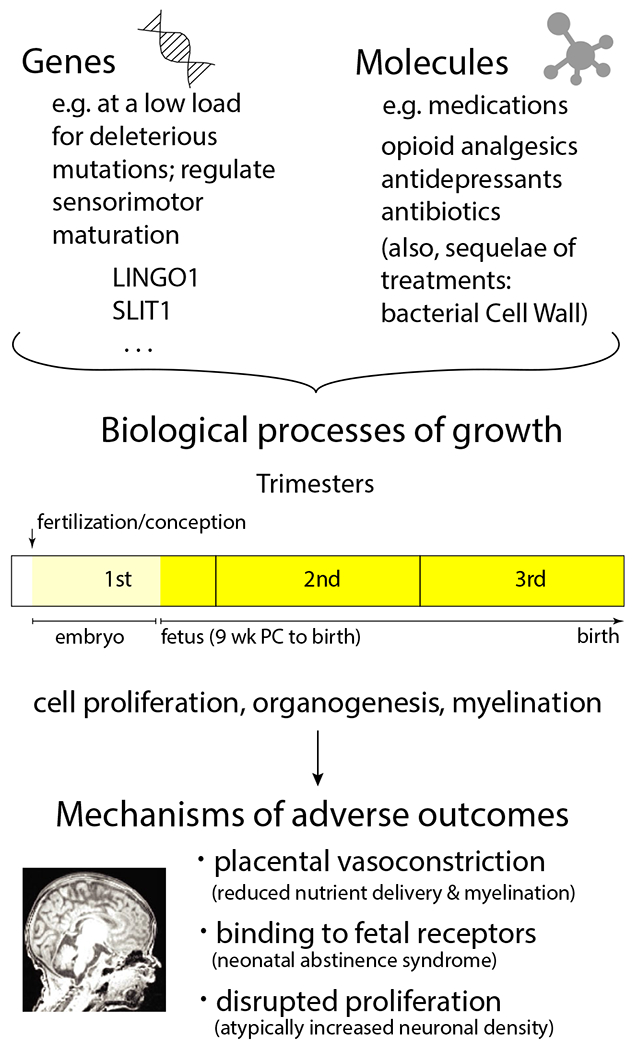
Vulnerability of embryonic and fetal growth and maturation during a 9-month pregnancy in the context of genetic and environmental (e.g., maternal medications) influences. The embryonic period corresponds to time from fertilization/conception to 8th week inclusive (equal to 10 weeks gestational age (GA) as counted from the first day of last normal menstrual period); the fetal period corresponds to week 9–37 or birth (11–39 GA or birth). The generation and differentiation of cells (neurons and glia, including oligodendrocytes, which regulate myelination in CNS) occur during the first trimester of pregnancy (1–13 GA). The cells proliferate, migrate to their positions, and mature and develop interconnections with other cells (Schacher, 1985). A period of heightened vulnerability is during organogenesis, approximately between 3 and 8 weeks after fertilization/conception (Sachdeva et al., 2009) (between 5 and 10 weeks GA), but teratogenic effect (such as congenital abnormality) may occur during the first 12 weeks after conception (Boskovic & Koren, 2005). Further, neuronal proliferation is greatest at mid-gestation (Rakic & Zecevic, 2000). Brain growth and maturation occurs during the second half of pregnancy and the third trimester (including e.g., due to myelination), and continues after birth. It important to understand the role of genetic vulnerability to antenatal maternal treatments in early sensorimotor development. LINGO1, leucine-rich repeat and immunoglobulin domain-containing protein; PC, postconception; SLIT1, Slit guidance ligand 1 (SLIT1)

In contrast, genetic vulnerability of the fetus to maternal treatments has not been investigated, even though genetic programming regulates fundamental neurobiological processes (e.g., differentiation, proliferation, migration, and myelination) that unfold during pregnancy (Figure 1). Deleterious aberrations in the genetic code may exacerbate the adverse contribution conferred by in utero pharmacological exposures on atypical growth and maturation of the baby.

For instance, genetic polymorphisms may make newborns more susceptible to medication-based NAS withdrawal symptoms after birth (Stover & Davis, 2015). Further, many likely gene-disrupting (LGD) de novo (DN) mutations (Iossifov et al., 2014) are targets of sensorimotor, motor, and vocal learning genes (Denisova, 2019). For example, a mutation of leucine-rich repeat and immunoglobulin domain-containing (LINGO1) gene, which controls stages of oligodendrocyte development and CNS myelination, could adversely impact idiosyncratic oligodendrocyte-neuronal interactions across cortical layers (Tomassy et al., 2016). Taken together, the consequences of antenatal pharmacological exposures may be exacerbated by disruptions in genes or clusters of genes that normally support key biological processes.

As normative sensorimotor functioning is fundamental to the postnatal cognitive development of the child (Adolph et al., 2018; Denisova, 2019b; Iverson et al., 2019), the goal of this work is to explore the role of genetic vulnerability of the fetus in the context of antenatal maternal treatments and maternal illness on sensorimotor functioning after birth. Specifically, a significant opportunity to reveal how contributions of in utero exposures (that may perturb different neurobiological processes prenatally) may converge on and reflect in a common developmental process postnatally is to study sensorimotor features during rest or sleep in individual infants or small subgroupings. Examined here are features of head movements from resting-state fMRI scans acquired during natural rest or sleep in 1- to 2-month-old infants with different genetic liabilities for autism and with different antenatal exposures.

There are important scientific reasons for wishing to use movement data from MRI scans to study development. Infant MRIs are obtained under well-controlled conditions that are comparable across scanners and across different laboratories and research groups worldwide, which is important given the challenges in obtaining usable and generalizable data with infants. Moreover, spontaneous head movements currently remain understudied despite early recognition for their importance in early sensorimotor development in infants (Denisova & Figurin, 1926). Movements of the limbs but not the head form the basis of the scheme of infant movements formalized by Prechtl in the 1960s and 1970s (Prechtl, 1974) and used to date. Historically, though, the first quantitative series of studies of sleeping infants and toddlers by Denisova and colleagues in the 1920s (Denisova & Figurin, 1926) formally described features of the movements of the head as well as the limbs and body, nearly 100 years ago. Incidentally, their study also marked the first published scientific account of rapid eye movements during infant sleep. Given this background, studying infant head movements from extant MRI scans can help close the gap in our knowledge on spontaneous head movements in infants during rest or sleep.

It is unknown whether subtle sensorimotor deficits during rest or sleep are associated with in utero exposure to medications in infants at high versus low genetic (familial) risk for developing autism spectrum disorders (ASD). One possibility is that regardless of whether or not an utero exposure was experienced, 1- to 2-month-old infants at a higher genetic risk (HR) for autism, who have an older biological sibling diagnosed with an ASD, would show the worst sensorimotor patterns relative to infants at low risk (LR). This “genetic primacy” prediction would be consistent with previous work showing that 1- to 2-month-old HR infants have the most atypically noisy head movements during sleep fMRI scans (Denisova & Zhao, 2017), and future ASD diagnoses at 3 years are predicted by atypically inflexible movements to different experimental conditions in 9- to 10-month-olds (Denisova, 2019b). However, another possibility is that genetic and environmental liabilities may interact to produce the most atypical sensorimotor outcomes. Would in utero exposure to different classes of drugs dissociate between movements of exposed versus non-exposed infants who are at a genetically higher (vs. lower) risk for developing ASD? Specifically, one prediction is that infants at HR for ASD with in utero psychotropic medications exposures would show “worse” motor features versus those of all other infants. The goal is to investigate whether several important maternal health variables before birth (in utero exposure to prescription medication, infection and fever during pregnancy, and maternal psychiatric diagnoses) map onto relevant neurobiological features of the infant immediately after birth: specifically, sensorimotor features of head movements during sleep rs-fMRI in 1- to 2-month-old infants at high or low risk for autism.

## 2 | METHODS

### 2.1 | Data

The data used in the preparation of this study were obtained from the National Institute of Mental Health (NIMH) Data Archive (NDA), formerly called the National Database for Autism Research, NDAR. NDA is a collaborative informatics system created by the National Institutes of Health to provide a national resource to support and accelerate research in mental health. Dataset identifier (along with the Submitter) is NDARCOL0002026 (Susan Bookheimer). The data used in the current study are fully de-identified in compliance with the U.S. Health Insurance Portability and Accountability Act (HIPAA) guidelines. Prior to being deposited in NDA and before commencement of any assessments and data collection procedures, signed written informed parental consent was obtained by original study investigators in accordance with U.S. 45 CFR 46 and guidelines laid down in the Declaration of Helsinki for participation; research protocols which included neuroimaging and clinical assessments were approved by the original study investigators local ethics committee at the University of California, Los Angeles (UCLA). The present study involved NDA data and did not involve research with human subjects or research with identifiable data. Analyses of these fully de-identified data in the current study were reviewed and approved by, and a waiver of informed consent was obtained from, the Institutional Review Boards of Columbia University Irving Medical Center and Teachers College, Columbia University.

The current study includes infant data (*N* = 56) acquired at the Autism Center of Excellence (ACE) at UCLA. This dataset comprises neurologically healthy infants (35 Males, M/21 Females F) meeting inclusion criteria of full-term birth (>36 weeks gestational age) and with birthweight over 3000 g. The neuroimaging dataset partially overlaps (1/3 new data) with datasets included in the Denisova and Zhao (2017) report. Importantly, the key inclusion criteria in the current study were a requirement that infants have associated data on maternal drug and health information: infants at 1–2 months of age with rs-fMRI data *and* for whom prescription drug information related to mother-driven prenatal risk factors was newly available via detailed infant and family medical histories. There were *N* = 26 infants (16 M/10 F) at high familial risk (HR) for developing Autism Spectrum Disorders (ASD), and *N* = 30 at low risk (LR) (19 M/11 F) satisfying this requirement. Familial autism risk is defined by virtue of the newborn baby having an older biological sibling diagnosed with ASD. This study examines, for the first time, the association between environmental, maternal risk factors during pregnancy and sensorimotor signatures of infants, as a function of the genetic, familial autism risk.

### 2.2 | Data preprocessing

**FIGURE 2:**
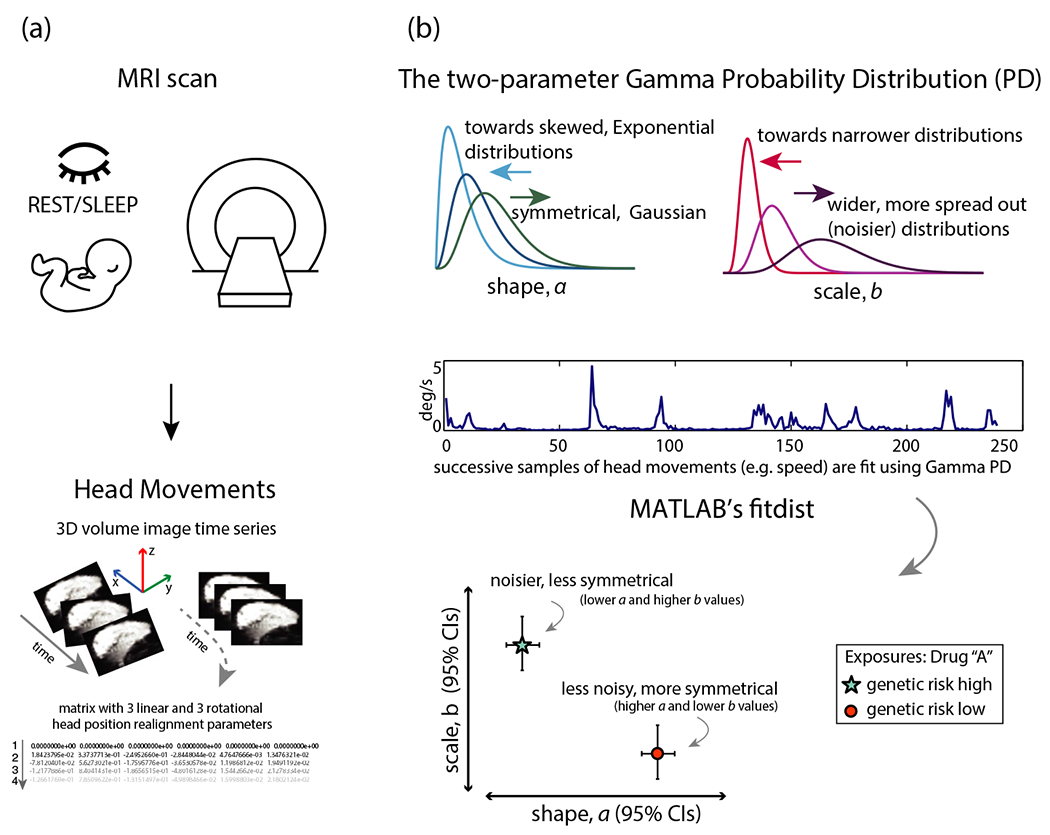
Head movements during infant MRI scans and data analysis path. (a) head movements are obtained from extant infant resting-state sleep MRI scans. The scans are acquired during rest or sleep, volume images are realigned, and the resulting time series are saved in a text file. (b) the path from movement data to fitted data. The *top panel* illustrates two parameters, shape (*a* parameter), and scale (*b* parameter), which characterize the gamma probability distribution (PD). The *middle panel* shows sample movement time series (speed shown). The *bottom panel* shows two parameter estimates of the gamma probability distribution (overhead movements), shape (*a* parameter), and scale (*b* parameter) for two hypothetical data points (genetic risk high and genetic risk low). Note that on the *x*-axis of the bottom panel, the leftward values indicate those tending towards more exponential distributions on the shape parameter (whereas the rightward values tend toward more symmetrical Gaussian distributions). On the *y*-axis, higher values indicate higher noise-to-signal levels on the scale parameter (lower values indicate lower noise-to-signal levels). In this scenario, relative to the data point labeled “genetic risk low,” the “genetic risk high” data point has higher values on the *y*-axis, which indicates higher noise-to-signal levels on the scale parameter. This data point also has more leftward values on the *x*-axis, which indicates a tendency towards less symmetrical, more Exponential distributions on the shape parameter (whereas the rightward values tend toward more Gaussian distributions). CIs, confidence intervals

Resting-state functional magnetic resonance imaging (rs-fMRI) data were acquired during natural sleep using a gradient-echo, echo-planar imaging (EPI) sequence (sampling rate: Time of Repetition, TR = 2,000 ms). There were 240 volumes per subject acquired during the sleep rs-fMRI scan. Specifically, acquisition parameters were as follows: [TR/TE: 2,000/28 ms, flip angle = 90°, FOV = 192 mm, 56 × 56 matrix, 34 axial 4 mm slices], with scan duration of 8 min (240 volumes). The preprocessing and analytical approach steps closely follow those in previously published work (Denisova, 2019c; Denisova & Zhao, 2017; Denisova et al., 2016), using free, open-source software. The EPI data were preprocessed to spatially realign the volume images (Friston et al., 1995; Friston et al., 1996) using SPM running under MATLAB (The MathWorks, Inc.), a process that generates rigid-body transformations as a time series, 3 linear (translations in the *x, y,* and *z* directions) and 3 rotational (pitch: about the *x*-axis, roll: about the *y*-axis, and yaw: about the *z*-axis) parameters (in radians; converted to degrees (*180/pi)). Movement time series were converted to speed using standard formulas (Denisova, 2019b; Denisova & Zhao, 2017) and filtered. The linear speed (based on linear realignment parameters) is expressed as mm/second; angular coordinates were Euler angles, and the angular speed (based on rotational realignment parameters) is expressed in deg/s. Figure 2a illustrates the concept of producing realignment parameters (the movements of the infant head during the MRI scan) to yield a time series for subsequent statistical analyses (“upcycling”) (Figure 2a).

### 2.3 | Analytical approach

Previous work with similar movement data obtained from fMRI or rs-MRI scans (Denisova & Zhao, 2017; Denisova et al., 2016) has shown that these data do not meet normality assumptions. Whether these movement time series violate the normality assumption was formally tested here using 3 tests, Kolmogorov-Smirnov (K-S), Lilliefors, and Jarque-Bera (all were two-tailed). This analysis established (13,449 speed data points) that data are not normally distributed (K-S, *p* < .001, Lilliefors, *p* = .001, Jarque-Bera, *p* = .001). The time series were fit using the Gamma distribution, a two-parameter family of continuous Probability Distributions (PDs), ranging from Gaussian (normally distributed, symmetrical) to Exponential (more random and noisy, heavy-tailed). The Gamma PD is appropriate to represent biological signals (Li et al., 2018; Maimon et al., 2009; Mochizuki et al., 2016). The Gamma PD does not require *a priori* assumptions about the signal’s underlying distribution (i.e., Gaussian or non-Gaussian). The probability density function (PDF) of the Gamma distribution is defined as $y=(x|a,b)=\frac{1}{b^{a}\text{Γ}(a)}x^{a-1}e^{\frac{-x}{b}},\text{for}\;x>0$, where Γ(∙) is the Gamma function (modeling sums of exponentially distributed random variables), and *a* and *b* are its *shape* and *scale* parameters, respectively.

The *top panel* in Figure 2b illustrates potential variations of the two parameters of the Gamma PD, shape, and scale. The shape (*a*) parameter represents information about the symmetry (or degree of skewness) of the distribution. When the shape parameter is small (tending toward 0), the distribution tends to be more Exponential (more skewed). On the other hand, when the shape value is large, distribution tends toward a more normal, symmetrical, or Gaussian distribution. The scale (*b*) parameter represents information about the *dispersion* in the data (i.e., noise to signal). Note that the *b* parameter is equivalent to the empirically estimated Gamma variance *σ*^2^ (defined as a*b^2^) divided by the mean *μ* (defined as a*b): a*b^2^/a*b. Considering both parameters, when dispersion is high, the distribution can look flatter and more symmetrical. A normal or a Gaussian distribution is symmetrical but can be relatively flat. This means that the values are not tightly clustered around the mean, and thus, there is more noise, or variability, in the data. Alternatively, the values can be tightly clustered around the mean, and in this case, the distribution would also look normal or Gaussian, but be very narrow and tall. Both parameters represent distinct information about statistical features of the data.

The *bottom panel* of Figure 2b illustrates the relationship between the two parameters with hypothetical data points due to exposure to “drug A”. Large *a* values (presented along the *x*-axis) indicate that the distribution is closer to the normal (Gaussian) distribution, whereas smaller values indicate a shift toward a more Exponential distribution. Small *b* values (*y*-axis) indicate a narrower, less spread out distribution (lower noise-to-signal levels), and large *b* values indicate a wider, more spread out distribution (higher noise-to-signal levels). In this example, relative to the data point labeled “genetic risk low,” the “genetic risk high” data point has higher values on the *y*-axis, which indicates higher noise-to-signal levels on the scale parameter. This data point has also has more leftward values on the *x*-axis, which indicates a tendency toward more Exponential distributions on the shape parameter (whereas the rightward values tend toward more Gaussian distributions) (see bottom panel of Figure 2b).

MATLAB’s fitdist function estimates 95% confidence intervals (CIs) for Gamma fits using the maximum likelihood estimation (MLE) procedure (https://www.mathworks.com/help/stats/fitdist.html). The functions and tools (e.g., the cftool) in the Statistics and Machine Learning and Curve Fitting Toolboxes in MATLAB were used for all statistical analyses. The analyses were performed on individual infant datasets, as well as pooled data across infant subgroupings pertaining to mother’s risk factors, separately for HR and LR infants. For the subgroup analyses, individual participant-level data were combined and a single distributional fit is performed on the time series (see below). MATLAB’s MLE-based fitdist algorithm successfully converged on a solution for every individual infant and every subgrouping for all fits reported. The following are details on forming different infant subgroupings related to important mother’s risk factors, information which was available for *N* = 56 unique infants. In particular, subgroupings derive from those infants with available infant medical history: available for 55 out of 56 infants, as well as from those with available family medical history (available for 47 of out of 56 infants; one infant with available family medical history did not also have available corresponding infant medical history).

### 2.4 | Maternal prescription medication (Rx)

The exposure status is confirmed for *N* = 55 infants. A total of *N* = 28 infants had a mother with a confirmed intake of a prescription medication during pregnancy (*N* = 14_HR_, *N* = 14_LR_). A total of *N* = 27 infants were not exposed in utero to any prescription medication (*N* = 11_HR_, *N* = 16_LR_).

Infants who were exposed to prescription medication were grouped into two groups: those with psychotropic and those with non-psychotropic medication exposure. Specifically, *N* = 8 had psychotropic exposure (*N* = 3_HR_; *N* = 5_LR_) and *N* = 20 had non-psychotropic exposure (*N* = 11_HR_; *N* = 9_LR_). In follow-up analyses, the psychotropic group was further broken down and examined by the specific psychotropic medication, as follows.

Specific *psychotropic* medications included different opioid analgesics or pain killers with opioid-type ingredients. A combination of acetaminophen and hydrocodone (the opioid-type ingredient), an opioid pain medication (Vicodin brand), or a combination of acetaminophen and oxycodone (the opioid-type ingredient), another opioid pain medication (Percocet), was taken by mothers of 4 infants, 2 from each group. Both HR infants were exposed to the combination of acetaminophen and hydrocodone (Vicodin), while both LR infants were exposed to the combination of acetaminophen and oxycodone (Percocet). Other psychotropic medication in this dataset includes antidepressant (selective serotonin reuptake inhibitor (SSRI) fluoxetine (Prozac)). Only one infant, from the LR group, was exposed to this drug. From this subgroup, a separate analysis examined HR and LR infants with confirmed in utero exposure to opioid analgesics, and an infant from the LR group with confirmed in utero antidepressant (fluoxetine/SSRI) exposure. To probe potential NAS-associated effects on motor functioning in these data, for all subgroups, above-median peaks which could represent small tremors in the movements were extracted and analyzed. Examples of *non-psychotropic* medications include 5HT3 receptor agonists, diabetic medication, and antibiotics. A separate analysis examined infants with confirmed antibiotic exposures to treat maternal infection (see below). In this sample, no infants had mothers with reported intake of anti-epileptic medications during pregnancy.

#### 2.4.1 | Prescription medication exposures varied across trimesters

The *psychotropic* medication exposures were reported for *N* = 8 infants (*N* = 3_HR_; *N* = 5_LR_). Overall, all *N* = 8 infants in this study with reported psychotropic medication exposures experienced these exposures during the 3rd trimester. Exposures were reported during each of 3 trimesters for *N* = 2 HR and *N* = 1 LR infants, during the 1st and 3rd trimesters for *N* = 1 LR infant, during the 2nd and 3rd trimester for *N* = 1 HR infant, and during the 3rd trimester only for *N* = 2 HR and *N* = 1 LR infant.

Note that in this psychotropic medication subgroup, several infants were exposed to opioid analgesics and fluoxetine (SSRI); their specific by-trimester exposures are as follows. Opioid analgesics exposures were during the 2nd and 3rd trimester for one HR infant, and during the 3rd trimester only for 3 infants (*N* = 2 LR and 1 HR). Fluoxetine (SSRI) exposure was for each of the 3 trimesters for one LR infant with this type of exposure.

The *non-psychotropic* medication exposures were reported for *N* = 20 infants (*N* = 11_HR_; *N* = 9_LR_). Non-psychotropic medication exposures were reported during each of 3 trimesters for *N* = 5 HR and *N* = 5 LR infants, during the 1rd trimester only for *N* = 2 HR and *N* = 2 LR infants, during the 2nd trimester only for *N* = 1 LR infant, during the 1st and 2nd trimesters for *N* = 1 HR and *N* = 1 LR infants, during the 2nd and 3rd trimesters for *N* = 1 HR infant and during the 3rd trimester only for *N* = 2 HR infants.

### 2.5 | Maternal psychiatric diagnoses (Dx)

Maternal health histories (including information about psychiatric diagnoses or conditions) were collected using parental questionnaires, according to procedures established by the assessment core at ACE UCLA. None of the mothers had a present or past diagnosis of ASD, as this was an exclusionary criteria during recruitment at ACE UCLA. This study asked if prenatally driven mental health conditions of the mother (including present or past history of a variety of conditions, such as attention-deficit hyperactivity disorder (ADHD), bipolar disorder, depression, obsessive-compulsive disorder (OCD), anxiety disorder, disrupted sleep, self-injuring behavior, learning disability, and eating disorder) could be linked to head movement signatures during the scan. Mothers’ information for these conditions is confirmed for a total of *N* = 47 infants (status is unknown for 1). Specifically, a total of *N* = 6 infants had a mother with confirmed multiple (at least 2) psychiatric conditions (*N* = 5_HR_, *N* = 1_LR_). A total of *N* = 9 had a mother with one psychiatric condition (*N* = 4_HR_, *N* = 5_LR_). A total of *N* = 31 infants had mothers with no psychiatric conditions (*N* = 12_HR_, *N* = 19_LR_).

### 2.6 | Maternal infection requiring antibiotics

A total of *N* = 13 infants had a mother who had an infection (e.g., a strep throat or a urinary tract infection, (UTI)) requiring antibiotics (*N* = 5_HR_, *N* = 8_LR_); the mother of *N* = 42 had no infection requiring antibiotics (*N* = 20_HR_, *N* = 22_LR_). Infection was reported to occur during each of 3 trimesters for *N* = 2 HR infants, during the 1st trimester only for *N* = 3 LR infants, during the 2nd trimester only for *N* = 1 LR infant, during the 1st and 2nd trimesters for *N* = 1 LR infant, and during the 3rd trimester only for *N* = 2 HR infants. Trimester information was not reported for *N* = 1 HR and *N* = 3 LR infants.

### 2.7 | Maternal fever higher than 101°F (38.3°C)

A total of *N* = 4 infants had a mother who experienced high fever (*N* = 2_HR_, *N* = 2_LR_); mothers of *N* = 50 did not experience high fever during pregnancy (*N* = 23_HR_, *N* = 27_LR_) (the status for this variable is known for *N* = 54 of out 55 infants with infant medical history; the status is unknown for 1). With regard to fever occurrence by trimester, the mothers of both HR infants reported having fever during the 1st trimester only. The mother of *N* = 1 LR infant reported two instances of fever: during the 1st and during the 3rd trimester; the mother of *N* = 1 LR infant reported having fever during the 2nd trimester only. The mothers of the four infants who experienced high fever did not report taking antipyretic medications to reduce fever symptoms.

## 3 | RESULTS

Seeking to discover whether in utero drug exposures during pregnancy, and other risk factors related to maternal health, such as a history of psychopathology, are associated with infants’ sensorimotor signatures during sleep rs-fMRI scans, this work examined, for the first time in the context of prenatally driven maternal risk factors, a large sleep rs-fMRI dataset with infants of a similar age (1- to 2-month-olds, *N* = 56; *N* = 26 infants at HR for autism) and for whom such prenatal variables were available.

**FIGURE 3:**
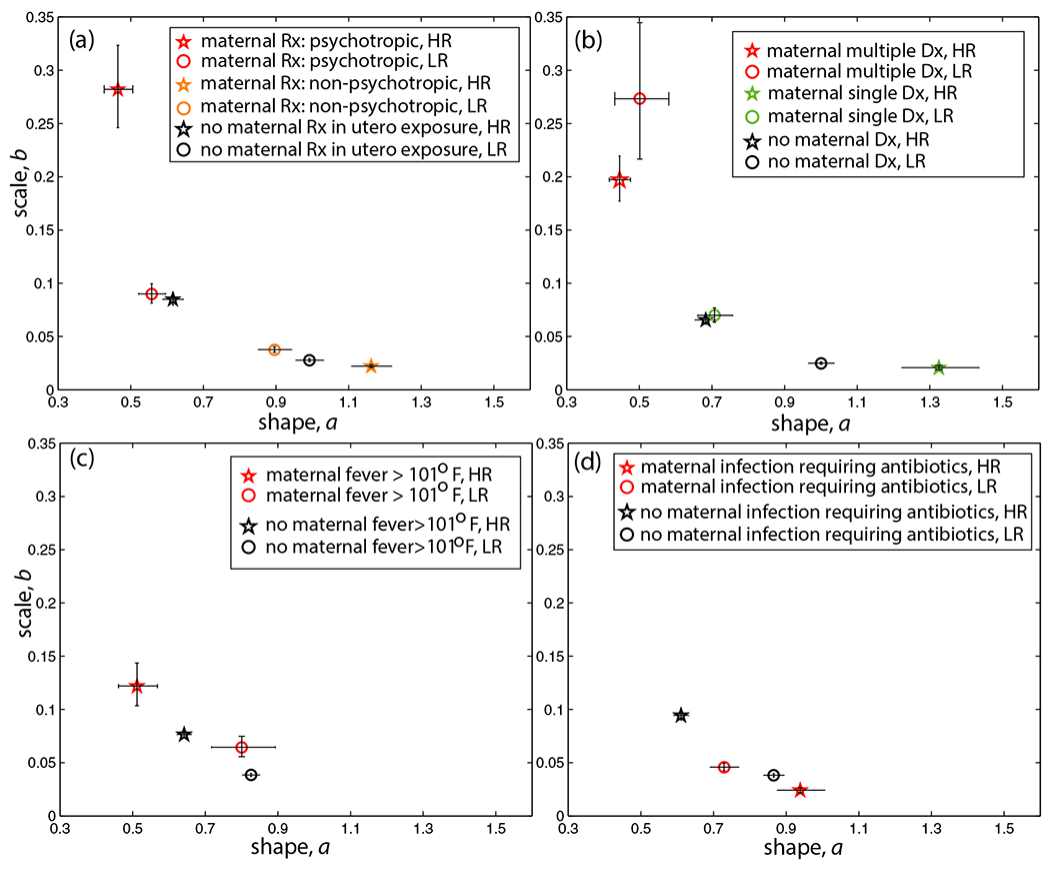
Linking features of infant continuous head movements during sleep rs-fMRI scan to maternal health during pregnancy, including intake of prescription medications, psychiatric diagnoses, and presence of fever or infections during pregnancy. The subgroupings are from a total of 56 infants (1–2 months of age), shown separately for infants at high genetic familial risk (HR) for developing autism and those at low risk (LR). The infants are subgrouped by (a) the prescription medication intake by the mother (psychotropic prescription medication, Rx: *N* = 3_HR_; *N* = 5_LR_; non-psychotropic Rx: *N* = 11_HR_; *N* = 9_LR_; No Rx: *N* = 11_HR_; *N* = 16_LR_), (b) maternal psychiatric conditions (two or more diagnoses, Dx: *N* = 5_HR_; *N* = 1_LR_; one Dx: *N* = 4_HR_; *N* = 5_LR_; no Dx: *N* = 12_HR_; *N* = 19_LR_), (c) maternal fever (high fever: *N* = 2_HR_; *N* = 2_LR_; no fever: *N* = 23_HR_; *N* = 27_LR_), and (d) maternal infection requiring antibiotics (infection: *N* = 5_HR_; *N* = 8_LR_; no infection: *N* = 20_HR_; *N* = 22_LR_). For each subgrouping, plotted are parameter estimates, shape (*a*) and scale (*b*), based on angular speed; the patterns are consistent for linear speed (Figure S2). (a) Sensorimotor signatures of psychotropically exposed infants have the highest noise-to-signal levels (higher *b* parameter estimate) and less symmetric distributions (lower *a* parameter estimate), tending towards Exponential (away from Gaussian) in both HR and LR infants, whereas non-psychotropic exposures are associated with the least noisy and most symmetrical sensorimotor signatures in HR relative to those of LR. (b) Exposures due to multiple maternal psychiatric diagnoses are characterized by parameter estimates with the highest noise-to-signal levels and decreased symmetry, tending towards the exponential distributions, across autism risk. (c) Maternal fever exposures for HR are characterized by significantly noisier and less symmetric parameter estimates relative to those of HR infants whose mothers did not report fever (this pattern is consistent for LR). (d) Maternal infection exposures for HR are characterized by atypically *lower* noise-to-signal levels and distributions tending towards Gaussian, relative to those of LR. Note that this ordering of parameter estimates for HR versus LR is consistent for non-psychotropic exposures shown in (a). Error bars denote 95% Confidence Intervals

An initial analysis confirmed the presence of individual heterogeneity of head movements during rs-fMRI scans acquired during sleep or rest (Figure S1). Further examination detected the contributions of both genetic and drug exposure status on movement, shown in Figure 3. The statistical features of the head movements are found to associate with various in utero “exposure” variables (parameter estimates based on angular speed, shown in Figure 3; consistent patterns are detected for linear speed in Figure S2). The different classes of drugs taken during pregnancy, a history of psychopathology during pregnancy, and variations in maternal immunity during pregnancy are associated with different effects on sensorimotor features during sleep in 1- to 2-month-old infants at high versus low familial autism risk (all data points are shown with 95% confidence intervals).

Among infants with no exposures of any kind and across the different subgroupings in Figure 3, a consistent pattern of genetic autism risk is seen such that HR infants with no exposures have significantly increased noise-to-signal levels and decreased symmetry (distributions tending toward exponential) relative to those of LR infants with no exposures (non-overlapping 95% CIs). This is a genetically driven “noise” effect, such that in non-exposed infants, non-exposed HR infants have noisier movements relative to non-exposed LR infants (Figure 3a–d).

The different levels of noise and symmetry are quantitatively linked to prescription medication drug classes taken by the mother during pregnancy (Figure 3a). The subgroupings include psychotropic medications (including opioid analgesics, SSRIs, and steroids), non-psychotropic medications (including antibiotics), or no prescription medication exposure. Importantly, movements with higher variability are detected in infants who were exposed in utero to psychotropic medications, which included exposures to opioid analgesics in both groups, and this pattern is significantly worse for infants at HR for ASD (Figure 3a). Non-psychotropic prescription medications differentially affect infant sensorimotor signatures relative to the no medication exposure group (Figure 3a; non-psychotropic vs. no medication exposure), such that atypically less noisy movements for non-psychotropic prescription medications are detected relative to those of non-exposed groups (Figure 3a).

These statistical features also associate with psychiatric disorders or conditions of the mother (Figure 3b), experiencing high fever during pregnancy (Figure 3c) or experiencing an infection requiring antibiotics during pregnancy (Figure 3d). Specifically, heightened maternal psychopathology is associated with noisier movements in both LR and HR subgroups (Figure 3b). Movements with higher variability are also detected in infants whose mothers had a high fever during pregnancy (Figure 3c), and this effect is significantly worse for HR infants. Atypically less noisy movements are detected when considering subgroups whose mother took antibiotics (non-psychotropic medication) while pregnant (Figure 3d), consistent with the pattern from non-psychotropic subgroupings (Figure 3a).

All infants with reported psychotropic medication exposures experienced these exposures during the 3rd trimester; the timing of this antenatal exposure is when one may expect NAS-like sequelae after birth. Thus, post hoc analyses looked for evidence of tremor-like features in psychotropically exposed (vs. non-psychotropically exposed) infants as a function of genetic risk. Analyses were repeated using above-median peaks (i.e., high movement points) in the data and revealed a similar pattern as the main results (Figure S3a–d). In particular, psychotropically exposed HR infants had the noisiest movement patterns relative to those of all other subgroupings, including relative to those of HR infants with non-psychotropic exposures (similar pattern was detected for LR infants) (Figure S3a). Further analyses focused on dissecting different Rx medications (opioid analgesics and fluoxetine (SSRI)), in this psychotropic subgrouping. HR infants who were exposed specifically to opioid analgesics had the noisiest movements relative to LR infants exposed to opioid analgesics, and the LR infant exposed to fluoxetine (SSRI) had noisier movements relative to non-exposed subgroupings (Figure S4; consistent pattern for parameter estimates based on above-median peaks data).

## 4 | DISCUSSION

Atypical sensorimotor functioning in newborn infants is driven not only by the heightened genetic risk of the infant for ASD, but also by their very early fetal exposures to maternal prescription medications. The key finding in the current study is that at 1–2 months after birth, different classes of medications (psychotropic vs. non-psychotropic) are associated with significantly atypical sensorimotor features. Infants exposed in utero to psychotropic medications have atypically noisier sensorimotor features relative to those of non-exposed infants, while infants exposed in utero to non-psychotropic medications have atypically less noisy statistical character of their sensorimotor movements relative to non-exposed infants.

One mechanistic way to understand these patterns is that atypical sensorimotor movements during sleep may suggest the presence of fragmented sleep in some infants, in part driven by prenatal factors (here, in the context of maternal health) and could affect memory consolidation processes and retention in particular (Simon et al., 2017) during a highly active period of sensorimotor learning for the developing nervous system (Adolph et al., 2018). Further, spontaneous movements (in particular, twitches, originating in the brainstem) in neonatal rats during sleep have been shown to contribute to building of sensorimotor maps in the CNS, arguably because such movements lack corollary discharge (Tiriac et al., 2014); atypical movements may adversely impact the developing CNS.

Surprisingly, it is not the case that HR infants have consistently noisier motor levels relative to those of LR infants. Instead, the current findings suggest an interplay between contribution from environmental in utero exposure to psychotropic medication and genetic liability for ASD on functionally important features during sensorimotor development for newborn infants. It matters whether or not HR infants were exposed in utero to psychotropic medications, which in this study includes exposures to opioid analgesics. Specifically, in utero exposure to psychotropic medication is associated with exacerbated sensorimotor signatures in HR infants, and, conversely, exposure to non-psychotropic medications (including antibiotics) associates with diminished movements. While psychotropic Rx-exposed HR infants (including Rx opioid analgesics-exposed HR infants, Figure S4) had the noisiest motor levels relative to LR infants, those HR infants who were exposed to *non*-Rx medications (Figure 3a,d), including *antibiotics,* had the least noisy levels relative to those of the other subgroupings. The data points for these HR subgroupings are in lower right-most location on the Gamma parameter plane.

This study further found that maternal mental health is an important contributor to early sensorimotor functioning. Heightened maternal psychopathology is associated with atypically noisier movements in infants regardless of their genetic autism risk. HR and LR infants whose mothers had multiple (2 or more) mental health disorders and conditions while pregnant had the noisiest motor features at 1–2 months after birth.

The psychopharmacological approaches to maternal psychopathology and pain management during pregnancy have developmental consequences for human offspring. Given that psychotropic medication-exposed infants, regardless of genetic risk, had atypically noisier sensorimotor features, it may be helpful to seek alternatives to pharmacology when possible. Awareness of the association detected here suggests the need for, and can inform, future development of alternatives to some prescriptions with opioid ingredients for pain management, such as those related to dental procedures, surgery, or to transient back pain. On the other hand, a complex situation exists when considering prescription psychotropics such as SSRIs, as these medications are prescribed to support maternal health (Sutter-Dallay et al., 2015).

While many researchers have noted severe adverse effects of psychotropic medications during early pregnancy, the consequences of exposures during the 3rd trimester are considered to be less harmful. However, previous research studies describing NAS symptoms (such as tremors due to psychotropic exposures) and indicating that these symptoms are thought to resolve a few weeks after birth did not use sensitive assays to measure motor functioning. Here, this study found that parameter estimates from high movement spikes (above-median peaks, which may constitute tremors) were consistent with the main analyses: Among the psychotropically exposed infants, all of whom had exposures during the 3rd trimester, 1- to 2-month-old HR infants had the noisiest movement signatures relative to non-psychotropically exposed HR infants.

The current findings suggest the possibility that NAS-like manifestations may persist for longer for some infants, especially if they are also at high genetic risk for autism. In particular, the effects detected in this study can be understood to reveal a subtle, persistent effect of NAS in HR group exposed to opioid analgesics. That the motor effects were detected also for opioid-analgesic-exposed LR infants argues that these subtle effects persist and are detectable using sensitive quantitative assays in infants at a genetically low risk for autism as well. Notably, this study used sensitive quantitative methods to show motor atypicalities even in infants with 3rd trimester exposures.

In the case of non-psychotropic medications such as antibiotics, infants’ sensorimotor signatures fall on the opposite location on the Gamma parameter plane relative to those of psychotropic and non-exposed infants, such that the signatures are atypically least noisy. Antibiotics fight infection but suppress the immune system, and given that at least some antibiotics or even the bacterial cell wall cross the placenta, these occurrences could disrupt homeostasis in the developing nervous system in the fetus and manifest as less abundant, less noisy movements postnatally. The character of these movements is very different from that of infants exposed to psychotropics, and is more similar to the movements of infants who were subgrouped by confirmed antibiotic use due to maternal infection, as well as the movements in the subgroupings due to reported maternal fever. The nature of the mechanistic process of how these exposures could reach the fetus is currently under study (Humann et al., 2016). Regardless of the exact mechanism, this study shows that maternal pharmacological therapies during pregnancy are associated with atypical sensorimotor movements in human offspring.

### 4.1 | Strengths and limitations, and future research directions

This study is the first to report associations between prenatal exposures to medications and subtle sensorimotor atypicalities by using sensitive objective techniques in a sample of 1- to 2-month-old infants with different genetic liabilities for autism. This study employs a user-friendly upcycling strategy according to the Open Science Infancy Protocol (Denisova, 2019a). This feature is important, as this protocol does not require collecting data from new human subjects and encourages innovative use of extant MRI data to address new questions of importance in the field of infancy research.

The current findings generate new research questions and directions. For example, sensorimotor features dissociate between non-psychotropic versus psychotropic medications and as a function of familial autism risk. A normative amount and character of spontaneous movements is very important for the developing nervous system in humans, and a pattern indicating either an excess (HR exposed to psychotropic (e.g., opioids)) or a lack thereof (HR exposed to non-psychotropic (e.g., antibiotics)), as the current study detects, is concerning. One question to probe in the context of etiology of ASD is whether these sensorimotor signatures revealed by genetic vulnerability to maternal treatments will differ depending on the biological sex of the younger baby in relation to the sex of the older sibling with ASD. For example, (Palmer et al., 2017) found significantly increased risk of recurrence ASD in HR infants who are male and whose older sibling is a female (surprisingly, the authors found lower risk for male HR infants with an older brother with ASD). It would be informative to explore in future work with larger samples whether the genetic, familial vulnerability, and maternal exposures on sensorimotor functioning detected in this study would interact with biological sex in the HR group to provide greater specificity for the risk of ASD development: that is, the sex of the baby infant and the sex of the older sibling already diagnosed with ASD.

Despite these important strengths, this work should be considered exploratory because of a small total number of infants available per exposure subgrouping and numerous molecules. For example, the number of infants in the key subgroupings includes *N* = 8 infants exposed to psychotropic prescription medication (*N* = 3_HR_ and *N* = 5_LR_; this number subsumes two infants in each risk group exposed to opioid analgesics) and *N* = 20 infants exposed to non-psychotropic medication (*N* = 11_HR_; *N* = 9_LR_). Also, within the non-psychotropic exposures, information on specific classes of antibiotics was not available. For example, information was not available on whether antibiotic belonged to a broad-class agent or narrow spectrum (narrow, not broad, are recommended for pregnant women (Nau et al., 2010)). Infection instances were not separated by UTIs and upper respiratory tract infections (URTs, research indicates that UTIs, but not URT exposures, are associated with psychopathology in child development (Dreier et al., 2018)). However, the sensorimotor consequences of non-psychotropic medications would have been even more atypical had there been more URT exposures. Multiple subgroupings of all infants were performed, given available data. Others have noted the difficulty in separating whether human offspring adverse outcomes are due to the underlying maternal illness, or the effects of treatment of the illness during pregnancy. Untreated illnesses are harmful for both the mother and the infant (Sutter-Dallay et al., 2015). Although none of the mothers diagnoses’ include ASD, the psychiatric diagnoses of the mother are heterogeneous. One possibility is that maternal sleep or anxiety could have cascading effects on psychiatric symptoms, for example, on ADHD. Including infants whose mothers have a particular disorder would enable for a more precise study of the role of the etiology of the given maternal psychiatric condition vis-à-vis biological risk and maternal mental health, on sensorimotor functioning in newborns.

Future research involving a large, international prospective study would be helpful, as it would enable conducting separate analyses on prenatal exposure to different medications with and without an underlying mental versus physical illness, and perhaps by trimester with infants at different levels of genetic risk. In future work, it would also be valuable to investigate the effectiveness of some of the counteractive actions to such exposures during pregnancy, including improvement in the sleep schedules of the parents (particularly the mother) and the older children, and to measure their influence on offspring sensorimotor outcomes. For these reasons, the teratogenicity of exposures in humans during pregnancy with respect to offspring functioning in the context of genetic vulnerability is an important, active research area in basic science in humans.

Within biological groups (high or low risk), the sensorimotor findings represent associations or correlations with antenatal exposures. Importantly, however, the current findings with human infants across risk groups go beyond a simple association or correlation, because the different risk groups (HR and LR) differ in the underlying biology. The biological, familial, genetic risk is a biological fact that distinguishes HR and LR infants and thus contributes to the nature of the sensorimotor features detected here. The situation is further complicated when considering that HR and LR groups differ in biology and could also potentially differ in other respects. For example, other differences, in addition to biology, may include higher parental stress in a family that already has a child with ASD. Taken together, an important factor when interpreting the overall findings, in particular across risk groups, is that the risk groups differ in biology, though additional factors may also play a role and merit future research.

## 5 | CONCLUSION

Head movements in early life have been typically used to reveal by cognitive psychologists *what infants know,* but recent findings indicate that head movements can also reveal critical biological information in early life about *how infants are.* Collaborative multi-disciplinary studies, including clinical trials in basic science in humans, are required to probe the specific contributions of environment (i.e., fetal exposures) versus genetics to the neurobiological sensorimotor processes unfolding during human prenatal and postnatal periods, and how perturbations of these processes may converge to produce atypical longer-term sensorimotor developmental consequences for the child. This study establishes for the first time that in newborns at 1–2 months after birth there are deleterious sensorimotor consequences associated with prenatal exposure to psychotropic medications, including analgesics containing an opioid-type ingredient. The consequences of these exposures are more severe in the presence of familial, genetic risk for autism. This finding suggests new research directions for the study of genetic vulnerability to antenatal exposure to maternal treatments during pregnancy and their contribution to infant development.

## Supplementary Material

Supplementary Information
