# Supplementary material for: Genetic vulnerability of exposures to antenatal maternal treatments in 1– to 2-month-old infants
Source: Infancy. Author manuscript; Available in PMC 2022 Dec 5. (PMC9721365; doi:10.1111/infa.12398)
Supplement: Supplementary Information [file NIHMS1851413-supplement-Supplementary_Information.docx]

**Supplementary Materials**

**Supplementary Figures**

**
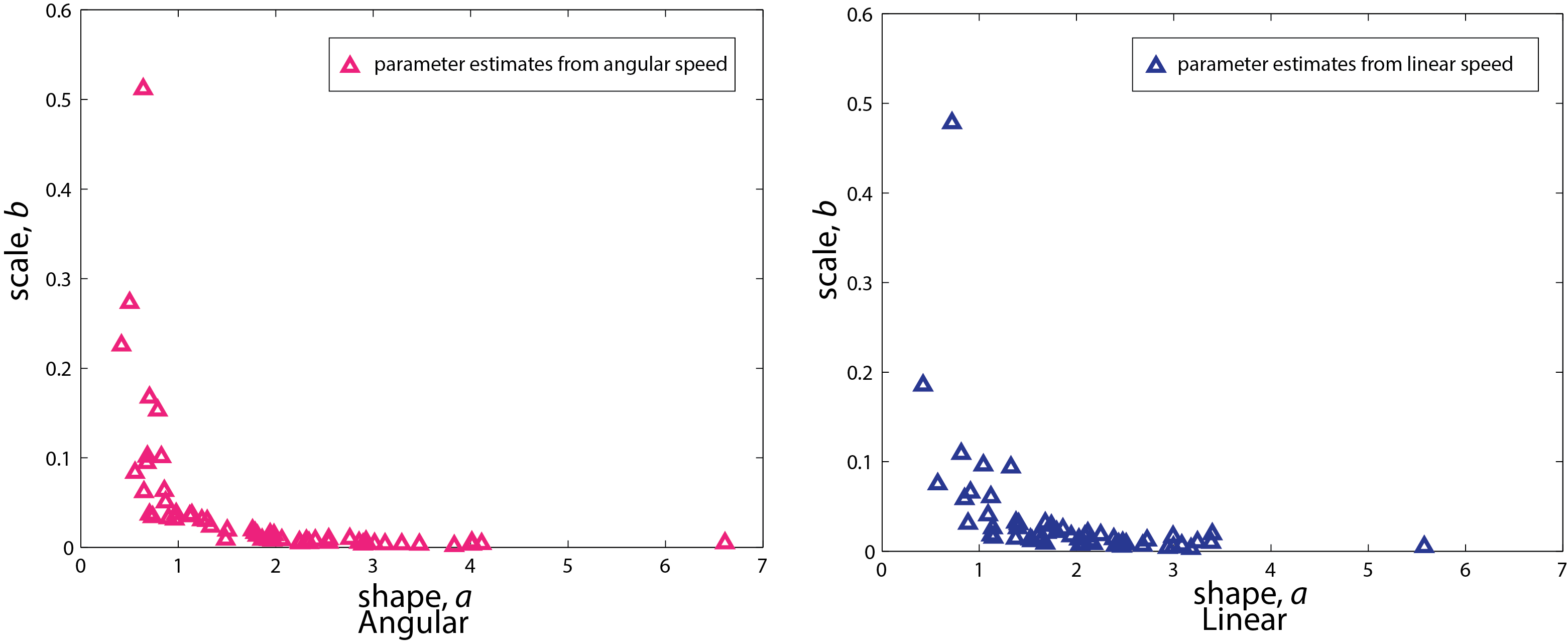
Supplementary Figure 1**. **Parameter estimates on the Gamma parameter plane for each individual infant (N=56 infants, 1-2 months of age).** Apparent individual diversity in head movement parameter estimates on the Gamma parameter plane for sleep rs-fMRI scans (similar patterns for angular and linear speeds). These data show substantial individual variation on both shape and scale parameters.

**
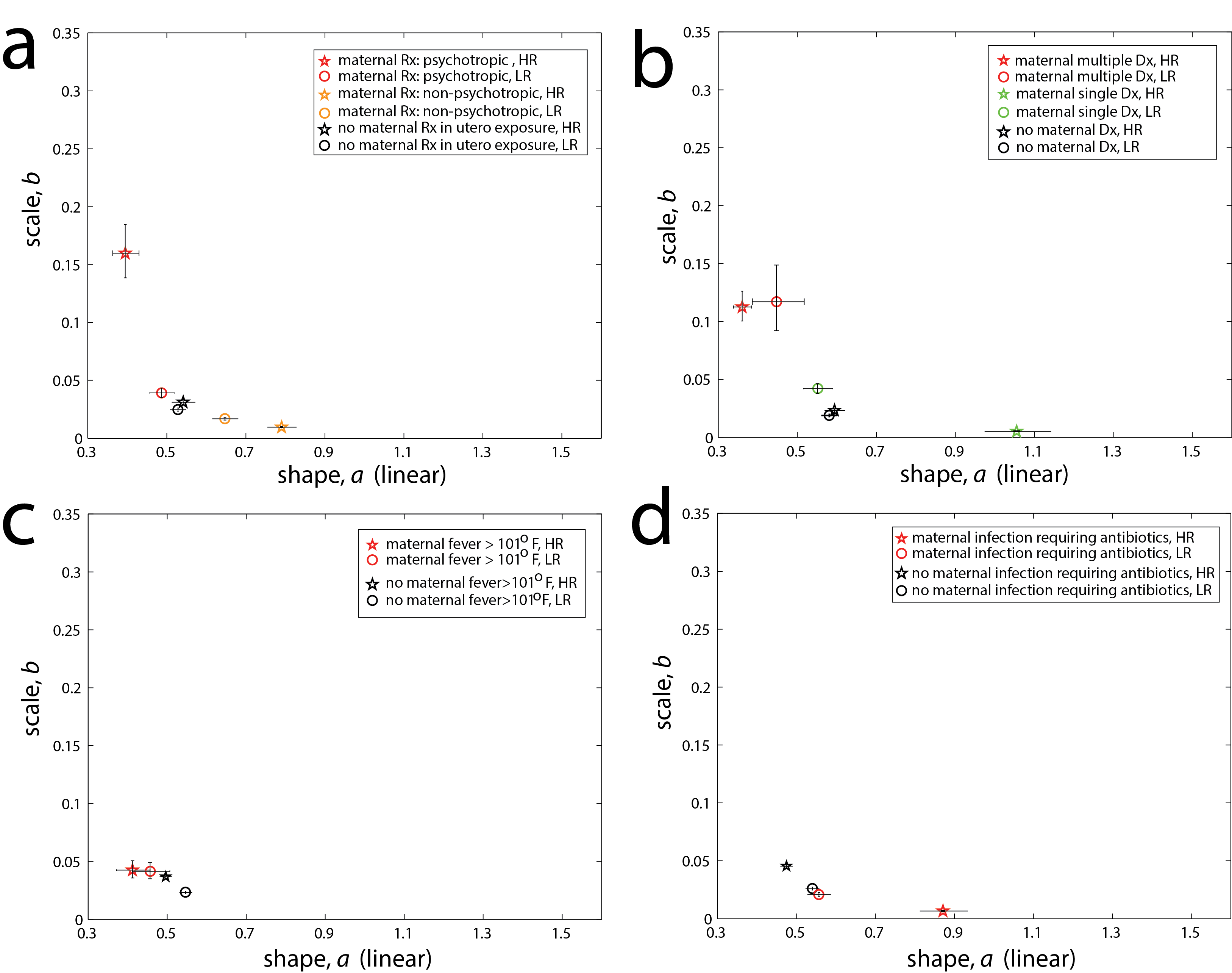
**

**Supplementary Figure 2. Linking features of head movements to maternal risk factors in infants with different genetic liabilities for autism: parameter estimates based on linear speed during sleep rs-fMRI (N=56 infants, 1-2 months of age).** Infant subgroupings are shown as a function of familial autism risk (High Risk, HR and Low Risk, LR). The subgroupings and Ns for each subgrouping are identical to those in **Figure 3** in the main text, which used angular speed. Infants were subgrouped by whether or not (**a**) the mothers took prescription medication (Rx) during pregnancy, (**b**) had psychiatric diagnoses (Dx), (**c**) experienced high fever, or (**d**) had infection requiring antibiotics. The parameter estimates for each of the subgroupings are shown with 95% Confidence Intervals.

**
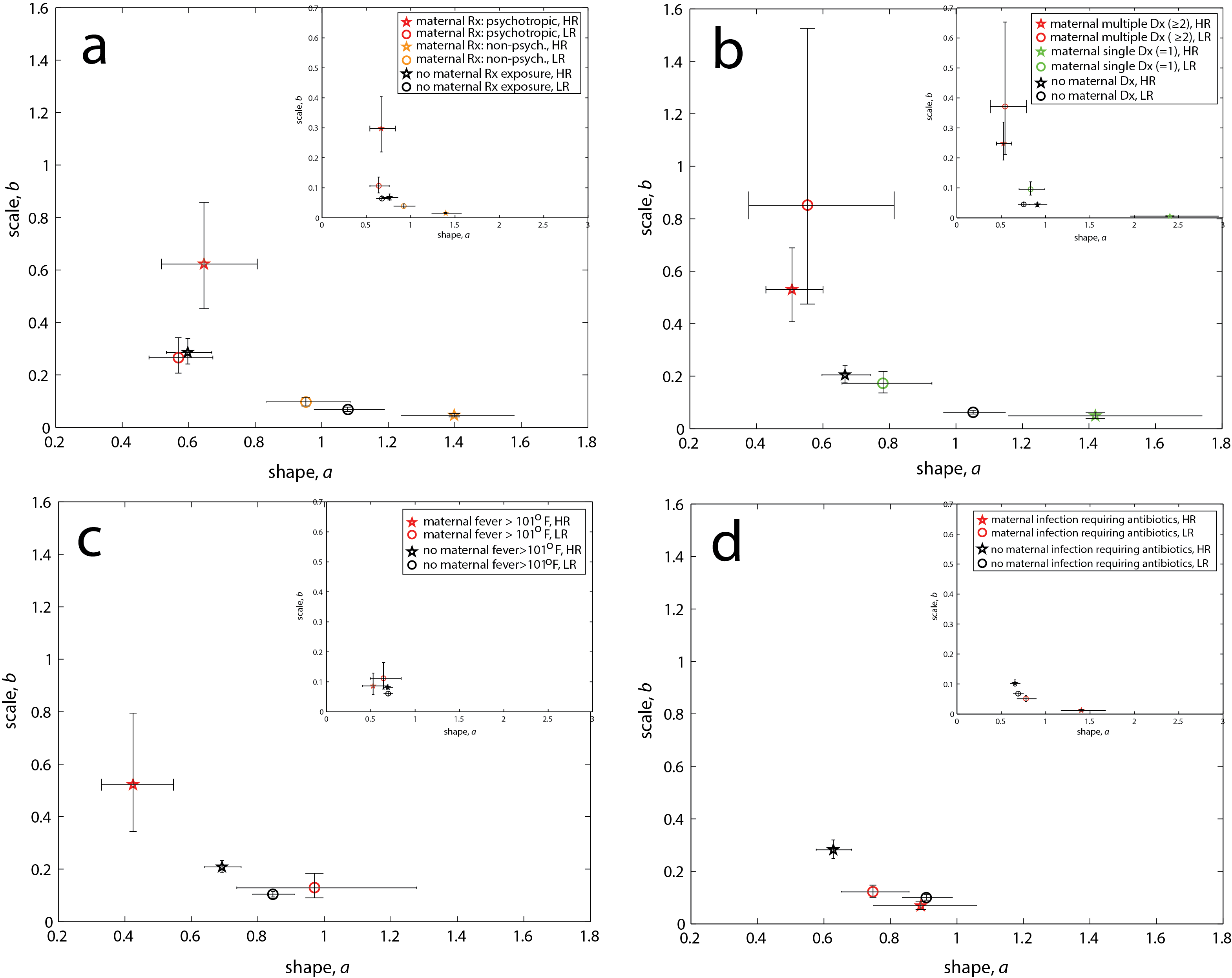
**

**Supplementary Figure 3. Parameter estimates based on *above-median* movement peaks during sleep rs-fMRI (N=56 infants, 1-2 months of age).** The subgroupings and Ns for each of the subgroupings are identical to those in **Figure 3** in the main text. Infants were subgrouped by whether or not (**a**) the mothers took prescription medication (Rx) during pregnancy, (**b**) had psychiatric diagnoses (Dx), (**c**) experienced high fever, or (**d**) had infection requiring antibiotics (High Risk, HR and Low Risk, LR). Shown are parameter estimates based on angular speed (insets show parameter estimates based on linear speed). The parameter estimates for each of the subgroupings are shown with 95% Confidence Intervals.

**
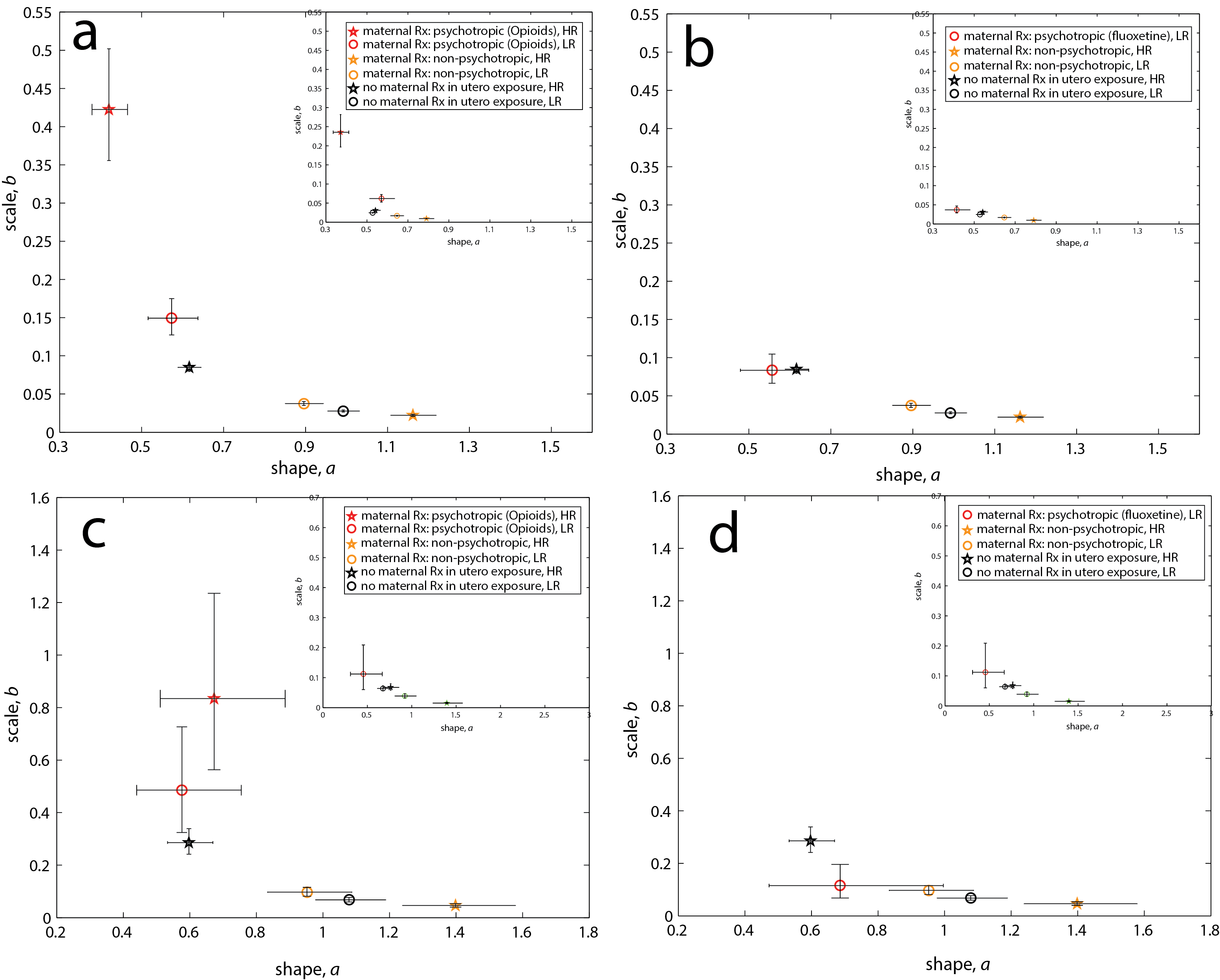
**

**Supplementary Figure 4. Rx psychotropic medication exposures to opioid analgesics and fluoxetine (SSRI) shown with non-psychotropic exposures and no exposure infant subgroupings in infants with different genetic liabilities for autism with movement data from sleep rs-fMRI scans (N=52 infants, 1-2 months of age).** The parameter estimates based on angular and linear (insets) speed are shown in **(a)** and **(b)**, and parameter estimates based on *above-median* movement peaks are in **(c)** and **(d)**. Infant subgroupings are shown as a function of familial autism risk (High Risk, HR and Low Risk, LR). The subgroupings are shown by distinct types of Rx psychotropic exposures, for (**a**) opioid analgesic medications Rx: N=2_HR_; N=2_LR_; and (**b**) an SSRI (fluoxetine) Rx: N=1_LR_ (no HR infants were exposed to SSRIs). The rest of the subgroupings and Ns (non-psychotropic Rx exposures and no psychotropic exposures) are included for comparison and are identical to those in **Figure 3** in the main text and **Supplementary Figure 3** (non-psychotropic Rx: N=11_HR_; N=9_LR_; No Rx: N=11_HR_; N=16_LR_). The parameter estimates for each of the subgroupings are shown with 95% Confidence Intervals.
